# Health-related quality of life and functionality in primary caregiver of surviving pediatric COVID-19

**DOI:** 10.3389/fpubh.2023.1117854

**Published:** 2023-06-20

**Authors:** Fernanda Martins, Fernanda T. Gonçalves, Marta Imamura, Daniela S. Barboza, Denise Matheus, Maria Fernanda B. Pereira, Heloisa H. S. Marques, Simone Correa-Silva, Marilia M. Montenegro, Thais T. Fink, Livia Lindoso, Vera Bain, Juliana C. O. A. Ferreira, Camilla Astley, Olivia M. Matsuo, Priscila Suguita, Vitor Trindade, Camila S. Y. Paula, Nadia Litvinov, Patricia Palmeira, Bruno Gualano, Artur F. Delgado, Magda Carneiro-Sampaio, Silvana Forsait, Vicente Odone-Filho, Leila Antonangelo, Linamara R. Battistella, Clovis A. Silva

**Affiliations:** ^1^Faculdade de Medicina, Instituto da Criança e do Adolescente, Hospital das Clínicas HCFMUSP, Universidade de São Paulo, São Paulo, SP, Brazil; ^2^Instituto de Medicina Física e Reabilitação, Hospital das Clínicas HCFMUSP, São Paulo, SP, Brazil; ^3^Laboratorio de Imunohematologia e Hematologia Forense (LIM-40), Departamento de Medicina Legal, Bioética, Medicina do Trabalho e Medicina Física e Reabilitação, Faculdade de Medicina, Hospital das Clínicas HCFMUSP, Universidade de São Paulo, São Paulo, SP, Brazil; ^4^Departamento de Medicina Legal, Bioética, Medicina do Trabalho e Medicina Física e Reabilitação, Faculdade de Medicina FMUSP, Universidade de São Paulo, São Paulo, SP, Brazil; ^5^Laboratório de Pediatria Clínica (LIM-36), Departamento de Pediatria, Faculdade de Medicina, Hospital das Clínicas HCFMUSP, Universidade de São Paulo, São Paulo, SP, Brazil; ^6^Faculdade de Medicina, Divisão de Reumatologia, Hospital das Clínicas, Universidade de São Paulo (HCFMUSP), São Paulo, SP, Brazil

**Keywords:** pediatric, children, adolescent, COVID-19, caregiver burden, quality of life, chronic disease, public health

## Abstract

**Objectives:**

To prospectively assess health-related quality of life (HRQoL), global functionality, and disability in primary caregivers of surviving children and adolescents after COVID-19.

**Methods:**

A longitudinal observational study was carried out on primary caregivers of surviving pediatric post-COVID-19 patients (*n* = 51) and subjects without COVID-19 (*n* = 60). EuroQol five-dimension five-level questionnaire (EQ-5D-5L) and 12-question WHO Disability Assessment Schedule 2.0 (WHODAS 2.0) were answered for both groups. The univariate regression analysis was carried out using SPSS (v 20) and significance was established at 5%.

**Results:**

The median duration between COVID-19 diagnosis in children and adolescents and longitudinal follow-up visits was 4.4 months (0.8–10.7). The median age of children and adolescents caregivers with laboratory-confirmed COVID-19 was similar to primary caregivers of subjects without laboratory-confirmed COVID-19 [43.2 (31.6–60.9) vs. 41.5 (21.6–54.8) years, *p* = 0.08], as well as similar female sex (*p* = 1.00), level of schooling (*p* = 0.11), social assistance program (*p* = 0.28), family income/month U$ (*p* = 0.25) and the number of household’s members in the residence (*p* = 0.68). The frequency of slight to extreme problems (level ≥ 2) of the pain/discomfort domain according to EQ-5D-5L score was significantly higher in the former group [74% vs. 52.5%, *p* = 0.03, OR = 2.57 (1.14–5.96)]. The frequency of disability according to WHODAS 2.0 total score was similar to those without disability and unknown (*p* = 0.79); however, with a very high disability in both groups (72.5% and 78.3%). Further analysis of primary caregivers of children and adolescents with post-COVID-19 condition (PCC) [*n* = 12/51 (23%)] compared to those without PCC [*n* = 39/51(77%)] revealed no differences between demographic data, EQ-5D-5L and WHODAS 2.0 scores in both groups (*p* > 0.05).

**Conclusion:**

We longitudinally demonstrated that pain/discomfort were predominantly reported in approximately 75% of primary caregiver of COVID-19 patients, with high disability in approximately three-quarters of both caregiver groups. These data emphasized the prospective and systematic caregiver burden evaluation relevance of pediatric COVID-19.

## Introduction

Pediatric-coronavirus disease 2019 (COVID-19) survivors may present persistent and disabling conditions after severe acute respiratory syndrome coronavirus 2 (SARS-CoV-2), named post-acute COVID-19 condition (PCC) (1–4).

PCC or “long COVID-19” manifestations is generally defined when any symptoms continued after 12 weeks of acute SARS-CoV-2 infection onset and cannot be justified by other conditions (5–8). The prevalence of PCC in pediatric populations ranges from 0.31% to 30% (1, 9, 10) and studies rarely included populations of children and adolescents with chronic preexisting conditions (3, 5).

In this regard, we recently showed that children and adolescents with SARS-CoV-2 infection patients had a prospective impact on health-related quality of life (HRQoL) parameters, specifically in physical and school domains and 23% of them had PCC (3). World Health Organization (WHO) suggest achieving family caregiver health outcomes as post-COVID condition research priorities (11). Only few results appoint a high caregiver burden during the acute phase of children and adolescents with COVID-19, evaluating HRQoL, anxiety, and depression tools, in Iran and Italy cohorts (12, 13).

It has been established that have a child or adolescent with any chronic health condition disrupt the family life in many ways mainly the psychological domain. However, this fact is not assessed and frequently receive little to no assistance or guidance during the medical treatment of the children or adolescent (14, 15).

To our knowledge, there is no report assessing concomitant HRQoL, global functionality, and disability in primary caregivers of children and adolescents with laboratory-confirmed COVID-19 survivors, as well as in PCC subjects. These issues are particularly important in a pediatric population with predominant chronic conditions in a tertiary healthcare facility. To improve care coordination of pediatric population we need to analyze the caregiver burden by mapping their health status and eventually indicating dedicated action plans for caregivers at local services or public policies.

Therefore, the present study aimed to assess longitudinal HRQoL, global functionality, and disability in primary caregivers of children and adolescents with laboratory-confirmed COVID-19 survivors and primary caregivers of children and adolescents without laboratory-confirmed COVID-19 (control group). These parameters were also compared between primary caregivers of COVID-19 patients with and without PCC.

## Methods

A longitudinal observational study was carried out on the primary caregiver of surviving pediatric post-COVID-19 patients from Instituto da Crianca e do Adolescente do Hospital das Clinicas da Faculdade de Medicina da Universidade de São Paulo (ICr HCFMUSP), São Paulo, Brazil which was one of the main COVID-19 reference hospitals in the biggest Latin American city. This study included a convenience sample from inpatient and outpatient treated in the hospital since the COVID-19 pandemic beginning. The inclusion criteria were as follows: symptomatic inpatients and outpatients, laboratory-confirmed SARS-CoV-2 infection, and age between <18 years. The exclusion criteria were subjects with asymptomatic disease and those who did not complete the forms as previously reported (3). EuroQol five-dimension five-level questionnaire (EQ-5D-5L) and 12-question World Health Organization Disability Assessment Schedule 2.0 (WHODAS 2.0) instrument were answered by *n* = 51 consecutive primary caregivers of surviving pediatric post-COVID-19 patients. The control group of primary caregivers was enrolled at the outpatient clinic of the same tertiary hospital and included *n* = 60 primary caregivers of pediatric subjects without laboratory-confirmed COVID-19. Additionally, we point that control group recruitment were paired taking into consideration children or adolescent age, sex and type of chronic health related condition. Data from the present study were collected between October 2020 to October 2021. The primary caregiver was defined by the person directly responsible for all the daily care of children and adolescents.

SARS-CoV-2 infection was measured by real-time reverse transcription-polymerase chain reaction (real-time RT-PCR) and antibody analysis. Real-time RT-PCR to assess SARS-CoV-2 RNA was performed on swab-collected nasopharyngeal and/or oropharyngeal samples in the Molecular Biology Laboratory of our tertiary health-care facility (16). Antibodies against S proteins from the coronavirus spike were carried out by two different methods: an enzyme-linked immunosorbent assay for anti-SARS-CoV-2 IgG antibodies and a rapid immunochromatographic assay for anti-SARS-CoV-2 IgM and IgG antibodies (17, 18).

Data for all subjects were prospectively inserted in Research Electronic Data Capture (REDCap) database of information at our university hospital (HCFMUSP). This report was approved by the Ethical Committee of our tertiary hospital (CAEE 4.889.659) and the written informed consent was obtained from all the participants and their parents/guardians.

### HRQoL, global functionality and disability instruments in primary caregivers

HRQoL was analyzed by EQ-5D-5L Brazilian Portuguese language (19). This self-evaluation tool is a generic, standardized measure of perceived health status. This tool included current information on the day visit and a detailed analysis of five dimensions: mobility, self-care (hygiene and dressing), usual activities (work, study, housework, family, and leisure activities), pain/discomfort, and anxiety/depression. Each dimension comprises a five-level version of the answer: no (level 1), slight (level 2), moderate (level 3), severe (level 4), and extreme problems (level 5).

WHODAS 2.0 global evaluates global functionality and disability in 12-question, covering six domains of functioning in the last 30 days: cognition, mobility, self-care, getting along, life activities, and participation. Each question was recorded from 1 (no difficulty) to 5 (extreme difficulty or cannot do). The sum of the total score ranges from 0 to 48. The results were presented as with disability, without disability, and unknown and categorized as mild (1–4), moderate (5–9), and severe disability (10–48). This tool was validated in the Brazilian Portuguese language (20).

### Demographic, anthropometric, and preexisting health condition data in children and adolescents

Demographic data in laboratory-confirmed COVID-19 children and adolescents comprised: current age, sex, and skin color (21). Body mass index was defined by body weight divided by the square of the body height and presented in units of kg/m^2^. Skin color was self-reported in a specific form – the options were white, black/brown and yellow. Preexisting pediatric chronic conditions in children and adolescents were categorized according to the duration of signs and symptoms more than 3 months, and diagnosis by physician scientific knowledge, valid methods, or instruments according to specific pediatric diagnostic criteria (22, 23). PASC or long COVID-19 manifestations were defined when clinical abnormalities continue after 12 weeks of the onset of acute COVID-19 diagnosis and cannot be justified by other acute or chronic conditions (3).

### Statistical analysis

Statistical analyses were carried out using SPSS software, version 20 (IBM Corporation, Armonk, NY, United States). A non-parametric test (Mann–Whitney test) or parametric test (Student’s *t*-test) was used for continuous variables and presented by median (minimum and maximum values) or mean ± standard deviation with an odds ratio (OR), respectively. Fisher’s exact or chi-square tests were used for categorical variables. The level of significance was established at 5%.

## Results

The median duration between COVID-19 diagnosis in children and adolescents and longitudinal follow-up visits was 4.4 months (0.8–10.7). PASC was observed in 12/51 (23%) of children and adolescents. The most frequently reported symptoms at longitudinal follow-up visits among children and adolescents with long-term PASC were headache in 6/12 (50%), fatigue in 6/12 (50%), dyspnea in 4/12 (30%), anxiety in 2/12 (16%), chest pain in 2/12 (16%), arthralgia in 1/12 (8%), and memory loss in 1/12 (8%).

The median of the current age of the primary caregivers’ group of children and adolescents with laboratory-confirmed COVID-19 was similar to primary caregivers of subjects without laboratory-confirmed COVID-19 [43.2 (31.6–60.9) vs. 41.5 (21.6–54.8) years, *p* = 0.08], as well as similar female sex (*p* = 1.00), level of schooling (*p* = 0.11), social assistance program (*p* = 0.28), family income/month U$ (*p* = 0.25) and the number of household’s members in the residence (*p* = 0.68) in both groups (Table 1). The median of the current age of children and adolescents in the group of primary caregivers of COVID-19 patients was significantly higher compared to the primary caregiver of subjects without laboratory-confirmed COVID-19 [14.6 (8.1–18.2) vs. 12.7 (2.1–18.9) years, *p* = 0.008], however with low OR = 1.16 (1.04–1.29). The other variables were similar in both groups: female sex (*p* = 0.53), ethnicity (*p* = 0.82), anthropometric data (*p* = 0.13), and preexisting pediatric condition (*p* = 0.61; Table 1).

**Table 1:** Primary caregivers of children and adolescents with laboratory-confirmed coronavirus disease 2019 (COVID-19) group compared to primary caregivers of children and adolescents without laboratory-confirmed COVID-19 (control group).

Variables	Primary caregivers of COVID-19 patients (*n* = 51)	Primary caregivers of control group (*n* = 60)	*p*	OR (CI 95%)
**Primary caregiver**				
**Demographic**				
Current age, years	43.2 (31.6–60.9)	41.5 (21.6–54.8)	0.08	1.04 (0.99–1.10)
Female sex	51 (100)	60 (100)	1.00	-
Level of schooling (*n* = 49)				
University	10 (19.6)	20 (33.4)	0.11	1.25 (0.95–1.64)
High school	24 (47.1)	30 (50)		
Middle school	8 (15.7)	4 (6.65)		
Elementary school	6 (11.8)	4 (6.65)		
Unknown	3 (5.9)	2 (3.3)		
Social assistance program	14 (43.8)	17 (32.1)	0.28	1.64 (0.66–4.07)
Family income/month U$	456 (0–2,283)	685 (0–4,035)	0.25	0.91 (0.78–1.07)
Number of household’s members in the residence	4 (2–6)	4 (2–8)	0.68	0.93 (0.69–1.27)
**Children and adolescents**				
**Demographic**				
Current age, years	14.6 (8.1–18.2)	12.7 (2.1–18.9)	0.008	1.16 (1.04–1.29)
Female sex	31 (61.8)	31 (52.7)	0.53	1.17 (0.71–1.93)
**Skin color**				
White	27 (52.9)	33 (55)	0.82	1.05 (0.69–1.60)
Black and brown	1 (41.2)	25 (41.7)		
Yellow	2 (3.9)	2 (3.3)		
Unknown	1 (2.0)	0 (0)		
**Anthropometric data**				
Body mass index, kg/m^2^	20.2 (13.7–39.5)	18.9 (13–32.5)	0.13	1.06 (0.98–1.14)
Pediatric preexisting condition			0.61	1.06 (0.85–1.32)
Immunologic	24 (47.1)	22 (36.7)		
Hepatic or renal	6 (11.8)	11 (18.3)		
Oncologic	6 (11.8)	6 (10.0)		
Other chronic condition	7 (13.7)	6 (10.0)		
Healthy	8 (15.7)	15 (25.0)		

Results are presented in *n* (%), median (minimum-maximum values) and mean ± standard deviation.

Table 2 illustrates EQ-5D-5L and WHODAS 2.0 scores in primary caregivers of children and adolescents with laboratory-confirmed COVID-19 compared to primary caregivers of children and adolescents without laboratory-confirmed COVID-19 (control group). The frequency of slight to extreme problems (level ≥ 2) of pain/discomfort domain by EQ-5D-5L score was significantly higher in the primary caregivers of children and adolescents with laboratory-confirmed COVID-19 compared to the other group [74% vs. 52.5%, *p* = 0.03, OR = 2.57 (1.14–5.96)]. No differences were evidenced in the other domains of EQ-5D-5L (mobility, self-care, usual activity, anxiety/depression). The frequency of disability according to WHODAS 2.0 total score was similar to those without disability and unknown (*p* = 0.79); however, with a very high disability in both groups (72.5% and 78.3%). No differences were shown in other WHODAS 2.0 score parameters in both groups (*p* > 0.05). We also performed EQ-5D-5L and WHODAS 2.0 scores evaluations from healthy subjects vs. those with chronic health condition but no difference between these two groups was founded *p* > 0.05 (data not showed).

**Table 2:** EuroQol 5-Dimension 5-Level (EQ-5D-5L) and World Hath Organization Schedule (WHODAS) 2.0 scores in primary caregivers of children and adolescents with laboratory-confirmed coronavirus disease 2019 (COVID-19) compared to primary caregivers of children and adolescents without laboratory-confirmed COVID-19 (control group).

Variables of primary caregiver	Primary caregivers of COVID-19 patients (*n* = 51)	Primary caregivers of control group (*n* = 60)	*p*	OR (CI 95%)
**EQ-5D-5L score (*n* = 50, *n* = 59)**				
Mobility			0.21	1.48 (0.80–2.75)
Level 1	38 (76.0)	51 (86.4)		
Level 2	6 (12.0)	5 (8.5)		
Level 3	6 (12.0)	2 (3.4)		
Level 4	0 (0)	1 (1.7)		
Level 5	0 (0)	0 (0)		
Slight to extreme problems (level ≥ 2)	12 (24.0)	8 (13.6)	0.21	2.01 (0.74–5.49)
Self-care				
Level 1	46 (92.0)	54 (91.5)	0.83	0.94 (0.53–1.67)
Level 2	2 (4.0)	2 (3.4)		
Level 3	0 (0)	1 (1.7)		
Level 4	2 (4.0)	1 (1.7)		
Level 5	0 (0)	1 (1.7)		
Slight to extreme problems (level ≥ 2)	4 (8.0)	5 (8.5)	1.00	0.94 (0.27–3.41)
Usual activity			0.83	0.94 (0.53–1.67)
Level 1	44 (88.0)	52 (88.1)		
Level 2	5 (10.0)	5 (8.5)		
Level 3	1 (2.0)	1 (1.7)		
Level 4	0 (0)	1 (1.7)		
Level 5	0 (0)	0 (0)		
Slight to extreme problems (level ≥ 2)	6 (12.0)	7 (11.9)	1.00	1.01 (0.30–3.02)
Pain/discomfort			0.12	1.37 (0.92–2.05)
Level 1	13 (26.0)	28 (47.5)		
Level 2	22 (44.0)	17 (28.8)		
Level 3	10 (20.0)	10 (16.9)		
Level 4	5 (10.0)	3 (5.1)		
Level 5	0 (0)	1 (1.7)		
Slight to extreme problems (level ≥ 2)	37 (74.0)	31 (52.5)	0.03	2.57 (1.14–5.96)
Anxiety/depression			0.31	1.21 (0.84–1.74)
Level 1	22 (44.0)	28 (47.5)		
Level 2	14 (28.0)	18 (30.5)		
Level 3	7 (14.0)	9 (15.3)		
Level 4	5 (10.0)	4 (6.8)		
Level 5	2 (4.0)	0 (0)		
Slight to extreme problems (level ≥ 2)	28 (56.0)	29 (52.5)	0.69	1.23 (0.58–2.65)
WHODAS 2.0 12 item total score (0–48) (*n* = 51, *n* = 60)	3 (0–20)	2 (0–21)	0.75	1.01 (0.94–1.07)
With disability	37 (72.5)	47 (78.3)	0.79	1.14 (0.43–2.94)
Without disability	9 (17.6)	13 (21.7)		
Unknown	5 (9.8)	0 (0)		
With disability (1–48)			0.53	0.85 (0.51–1.41)
Mild (1–4)	21 (56.8)	25 (41.7)		
Moderate (5–9)	8 (21.6)	8 (13.3)		
Severe (10–48)	8 (21.6)	14 (23.3)		

Results are presented in *n* (%), median (minimum-maximum values) and mean ± standard deviation.

Further analysis of primary caregivers of children and adolescents with PCC [12/51 (23%)] compared to those without PCC [39/51 (77%)] revealed no differences between demographic data, EQ-5D-5L and WHODAS 2.0 scores in both groups (*p* > 0.05, Table 3).

**Table 3:** Demographic, EuroQol 5-Dimension 5-Level (EQ-5D-5L) and World Hath Organization Schedule (WHODAS) 2.0 scores in primary caregivers of children and adolescents with PCC compared to without PCC.

Variables of primary caregiver	Primary caregivers of COVID-19 patients with PCC (*n* = 12)	Primary caregivers of COVID-19 patients without PCC (*n* = 39)	*p*	OR (CI 95%)
**Demographic**				
Mother current age, years	40.22 (32.2–53.4)	44.9 (31.7–60.9)	0.17	0.94 (0.85–1.03)
Mother’s level of schooling			0.60	0.87 (0.56–1.40)
University	4 (33.3)	6 (15.4)		
High school	2 (16.7)	22 (56.4)		
Middle school	4 (33.3)	4 (10.3)		
Elementary school	2 (16.7)	4 (10.3)		
Unknown	0 (0)	3 (7.7)		
Social assistance program	3 (37.5)	11 (45.8)	0.71	0.71 (0.13–3.66)
Family minimum wages/month	2.2 (1–10)	2 (0–10)	0.24	1.18 (0.89–1.56)
Number of household’s members in the residence	4 (3–6)	4 (2–6)	0.06	1.79 (0.98–3.30)
**EQ-5D-5L score**				
Mobility			0.19	0.30 (0.05–1.82)
Level 1	10 (90.9)	28 (71.8)		
Level 2	1 (9.1)	5 (12.8)		
Level 3	0 (0)	6 (15.4)		
Level 4	0 (0)	0 (0)		
Level 5	0 (0)	0 (0)		
Slight to extreme problems (level ≥ 2)	1 (9.1)	11 (28.2)	0.25	0.25 (0.02–1.65)
Self-care			1.00	---
Level 1	11 (100)	35 (89.8)		
Level 2	0 (0)	2 (5.1)		
Level 3	0 (0)	2 (5.1)		
Level 4	0 (0)	0 (0)		
Level 5	0 (0)	0 (0)		
Slight to extreme problems (level ≥ 2)	0 (0)	4 (10.2)	0.56	---
Usual activity			0.65	0.63 (0.08–4.66)
Level 1	10 (90.9)	34 (87.2)		
Level 2	1 (9.1)	4 (10.3)		
Level 3	0	1 (2.5)		
Level 4	0	0		
Slight to extreme problems (level ≥ 2)	1 (9.1)	5 (12.8)	1.00	0.68 (0.05–18.9)
Pain/discomfort			0.35	0.69 (0.31–1.51)
Level 1	4 (36.4)	9 (23.1)		
Level 2	4 (36.4)	18 (46.2)		
Level 3	3 (27.3)	7 (17.9)		
Level 4	0 (0)	5 (12.8)		
Level 5	0 (0)	0 (0)		
Slight to extreme problems (level ≥ 2)	7 (63.6)	30 (76.9)	0.44	0.52 (0.14–1.92)
Anxiety/depression			0.22	0.64 (0.32–1.31)
Level 1	7 (63.6)	15 (38.5)		
Level 2	2 (18.2)	12 (30.8)		
Level 3	1 (9.1)	6 (15.4)		
Level 4	1 (9.1)	4 (10.3)		
Level 5	0	2 (5.1)		
Slight to extreme problems (level ≥ 2)	4 (36.4)	24 (61.5)	0.17	0.36 (0.10–1.51)
WHODAS 2.0 12 item score (0–48)	4 (1–19)	4 (0–21)	0.88	0.99 (0.86–1.13)
With disability	8 (66.7)	29 (74.4)	0.48	2.21 (0.24–20.35)
Without disability	1 (8.3)	8 (20.5)		
Unknown	3 (25.0)	2 (5.1)		
With disability (1–48)			0.93	0.96 (0.36–2.52)
Mild (1–4)	4 (50.0)	17 (58.6)		
Moderate (5–9)	3 (37.5)	5 (17.2)		
Severe (10–48)	1 (12.5)	7 (24.1)		

Results are presented in *n* (%), median (minimum-maximum values) and mean ± standard deviation, PCC, post-COVID-19 condition.

## Discussion

We prospectively demonstrated that pain or discomfort was predominantly reported in 75% of female caregivers after pediatric COVID-19 diagnosis. We also observed a high disability impact in approximately three-quarters of both primary caregivers with and without COVID-19 and anxiety/depression in more than 50% of both groups.

During the COVID-19 pandemic, our pediatric tertiary healthcare facility installed a multidisciplinary and multispecialty outpatient clinic for longitudinal and simultaneous studies for children and adolescents COVID-19 survivors’, such as HRQoL, cardiovascular and pulmonary assessments that were recently reported (3, 24, 25). The present study focused on primary caregivers of these children and adolescents, evaluating the caregiver burden in a tertiary health care facility from a low-income country.

The strong point of this observational study was the inclusion of primary caregivers of children and adolescents with laboratory-confirmed SARS-CoV-2 infection (by RT-PCR and sorology), with a predominance of pediatric chronic conditions subjects (22, 26). Another strength was the inclusion of a control group, including primary caregivers of children and adolescents without laboratory-confirmed SARS-CoV-2 infection at study entry, balanced by age, sex, level of schooling, social assistance program, family income/month, and the number of household’s members in the residence, since these parameters are a well-known factor that impacts HRQoL in adults (27). Another advantage of the present study was the simultaneous analysis of HRQoL, global functionality, and disability in primary caregivers. Indeed, the same self-reported tools have been used during acute and chronic adult COVID-19 studies (28, 29). However, we did not collect real-time RT-PCR or antibody tests to investigate previous SARS-CoV-2 infections in caregivers.

We extended the previous study and showed that pain or discomfort occurred in most young adults’ caregivers of children and adolescents COVID-19 survivors. These findings suggest that these issues might be related to SARS-CoV-2 infection. The marked persistent pain and/or discomfort symptoms in caregivers of children and adolescents with COVID-19 can be subsequent to musculoskeletal involvement and possibly related to muscle weakness, fatigue, chronic pain, and/or arthralgia (30, 31). We hypothesized that those young female caregivers were affected by SARS-CoV-2 and had persistent symptoms probably concomitant with our pediatric patients, as reported by other studies (30, 32).

Of note, disability was observed in more than three-quarters of both primary caregivers from children and adolescents with or without COVID-19. In addition, more than 40% of both our groups with young age had moderate to severe disabilities according to this instrument. Reinforcing the relevant caregiver burden of the present study, normative populational data of total WHODAS 2.0 12 item score showed that the majority of subjects with similar age of the present study (bracket age from 35 to 54 years old) had a mild disability (33).

In addition, anxiety and depression, according to the EQ-5D-5L score, occurred in more than 50% of both groups. This point seems unrelated to SARS-CoV-2 infection and may be explained by the COVID-19 pandemic that increased psychopathology in young people who spent hours per day assessing information about the outbreak (34). The mother’s fear of new SARS-CoV-2 infection, underlying disease activity, or complication related to immunosuppressive drugs may also contribute to this finding (27, 35). Furthermore, none of our children and adolescents of the present study were vaccinated at the time of this study which may also have contributed to these mental health abnormalities during this pandemic time.

PCC was not related to any impact on HRQoL, global disability, and functionality in our study. The small sample of PASC observed herein may have contributed to this result.

In conclusion, we longitudinally demonstrated that pain/discomfort were predominantly reported in approximately 75% of female caregivers of COVID-19 patients, with high disability frequency in approximately three-quarters of both caregiver groups. These data emphasized the prospective and systematic caregiver burden evaluation relevance of pediatric COVID-19 suggesting that is important to analyze the caregiver burden by mapping their health status during health care management of pediatric population. Create evidences about caregivers is crucial if we intend to implement a full and integrated lifelong care for chronic health conditions in a pediatric population. We intend to contribute to initial highlights about caregiver consequences in COVID-19 pediatric population mainly for PCC.

## Study limitations

The present observational report has some limitations. We did not include clinical and laboratorial data from primary caregivers since we designed the study to assess several outcomes in children and adolescents after COVID-19 diagnosis. This study also included a cohort from only one healthcare pediatric facility at the largest hospital in Latin America and an analysis of the first visit after the median of 4 months of acute COVID-19 diagnosis. Therefore, long-term caregiver burden studies will be required, including additional visit evaluations at 12 months.

## Data availability statement

The raw data supporting the conclusions of this article will be made available by the authors, without undue reservation.

## Ethics statement

The studies involving human participants were reviewed and approved by the Ethical Committee of Hospital das Clínicas HCFMUSP, Faculdade de Medicina, Universidade de São Paulo (CAAE 37460620.8.0000.0068). The patients/participants provided their written informed consent to participate in this study.

## Author contributions

FM, FG, MI, DB, DM, MP, HM, SC-S, MM, TF, LL, VB, JF, CA, OM, PS, VT, CP, NL, PP, BG, AD, MC-S, SF, VO-F, LA, LB, and CS contributed substantially to the conception and design of the study and to the analysis and interpretation of data. All authors contributed to the article and approved the submitted version.

## Funding

This study was supported by grants from Conselho Nacional de Desenvolvimento Científico e Tecnológico (CNPq 304984/2020-5 to CS), Fundação de Amparo à Pesquisa do Estado de São Paulo (FAPESP 2015/03756-4 to CS), Núcleo de Apoio à Pesquisa “Saúde da Criança e do Adolescente” da USP (NAP-CriAd) to CS, and HCFMUSP with funds donated by NUBANK under the #HCCOMVIDA scheme supported this study (to MM and SC-S).

## Conflict of interest

The authors declare that the research was conducted in the absence of any commercial or financial relationships that could be construed as a potential conflict of interest.

## Publisher’s note

All claims expressed in this article are solely those of the authors and do not necessarily represent those of their affiliated organizations, or those of the publisher, the editors and the reviewers. Any product that may be evaluated in this article, or claim that may be made by its manufacturer, is not guaranteed or endorsed by the publisher.
